# Disseminated Pulmonary Coccidioidomycosis Presenting as a Wedge-Shaped Necrotic Mass Mimicking Malignancy

**DOI:** 10.7759/cureus.116066

**Published:** 2026-09-10

**Authors:** Tanveer Singh, Sharanya Thiagarajan, Igor Gorodniuk, Armine Martirosyan, Firas Alhalaibeh

**Affiliations:** 1 Internal Medicine, Sierra View Medical Center, Porterville, USA

**Keywords:** acute respiratory distress syndrome (ards), amphotericin b nephrotoxicity, coccidioidal meningitis, coccidioidomycosis, diabetes mellitus, disseminated coccidioidomycosis, fungal pneumonia, lung mass, pulmonary coccidioidomycosis, valley fever

## Abstract

Coccidioidomycosis is an endemic fungal infection of the southwestern United States that may rarely produce mass-like pulmonary abnormalities mimicking malignancy. We describe a 70-year-old Hispanic woman with poorly controlled type 2 diabetes mellitus (HbA1c >14%) who presented after three days of generalized weakness, nonproductive cough, abdominal pain, nausea, vomiting, and progressive difficulty ambulating. CT demonstrated a 7.3 × 5.4 cm wedge-shaped necrotic right middle lobe mass with multiple bilateral pulmonary nodules. CT-guided biopsy of the mass demonstrated granulomatous inflammation with fungal elements, while subsequent serologic testing and bronchoalveolar lavage PCR supported pulmonary coccidioidomycosis. Her hospitalization was complicated by acute respiratory distress syndrome requiring invasive mechanical ventilation, prone positioning, and neuromuscular blockade; non-liposomal amphotericin B-associated acute tubular necrosis requiring hemodialysis; ventilator-associated *Klebsiella pneumoniae* pneumonia; and persistent encephalopathy with multifocal MRI abnormalities highly suggestive of central nervous system dissemination despite nondiagnostic cerebrospinal fluid studies. This case highlights how pulmonary coccidioidomycosis can mimic malignancy. It illustrates the multifactorial deterioration that may occur in susceptible hosts, emphasizing early tissue diagnosis, careful interpretation of suspected dissemination, and individualized antifungal and critical care management.

## Introduction

Coccidioidomycosis, caused by *Coccidioides immitis* and *Coccidioides posadasii*, is a dimorphic fungal infection endemic to arid regions of the southwestern United States, particularly California and Arizona [1]. Infection occurs through inhalation of airborne arthroconidia from disturbed soil, with incidence influenced by environmental factors such as rainfall followed by drought, a pattern often described as the “grow and blow” phenomenon [1].

The reported incidence has increased substantially over recent decades, with approximately 20,000 cases reported annually in the United States; however, the true burden is estimated to be 10-18 times higher, reflecting significant underdiagnosis [2,3]. The observed increase is likely multifactorial and may reflect environmental and climatic changes, population growth and exposure within endemic areas, and improved recognition and surveillance rather than a single mechanism [2,3]. In California, incidence has risen dramatically, particularly in the Central Valley, where some counties report rates exceeding 200 cases per 100,000 population [3]. While most infections are mild or asymptomatic, about 5-10% of patients develop complications, and dissemination occurs in about 0.5-2% of cases, often with substantial morbidity and mortality [1,4]. Patients with impaired cellular immunity and other host susceptibility factors, including poorly controlled diabetes mellitus, are at increased risk for progressive disease [1].

Radiographically, pulmonary coccidioidomycosis most commonly manifests as nodules, consolidation, or cavitary lesions. However, mass-like necrotic lesions are rare and may closely mimic malignancy, creating substantial diagnostic uncertainty and potential delays in appropriate management [5]. When such a lesion is necrotic and wedge-shaped, the differential expands further to include vascular and embolic processes, making tissue diagnosis particularly important.

This report describes a case of pulmonary coccidioidomycosis presenting as a large wedge-shaped necrotic lung mass initially concerning for metastatic malignancy, followed by acute respiratory distress syndrome (ARDS) and multisystem critical illness with strongly suspected central nervous system (CNS) dissemination.

This case was previously presented as a case report poster at the 2025 CHEST Annual Meeting (October 20, 2025) during the session “Chest Infections Case Report Posters (E).”

## Case presentation

A 70-year-old Hispanic woman with a history of hypertension and poorly controlled type 2 diabetes mellitus presented with three days of generalized weakness, nonproductive cough, abdominal pain, nausea, vomiting, and progressive difficulty ambulating. The history was supplemented by information from the family, who reported a similar self-limited episode approximately one month earlier followed by progressive functional decline and unintentional weight loss. No similar illness among household contacts was documented in the available history.

On presentation, she was tachycardic at 108 beats/min, hypertensive at 190/81 mmHg, and had an oxygen saturation of 94% on room air. Laboratory evaluation demonstrated a white blood cell count of 18.5 × 10⁹/L, sodium 124 mmol/L, chloride 92 mmol/L, calculated serum osmolality 259 mOsm/kg, glucose 233 mg/dL, HbA1c >14%, blood urea nitrogen 21 mg/dL, creatinine 1.1 mg/dL, and lactate 1.1 mmol/L (Table 1). There was no diabetic ketoacidosis. Her hypotonic hyponatremia occurred in the setting of nausea, vomiting, and poor oral intake and was initially managed with intravenous fluids.

**Table 1:** Pertinent laboratory findings on admission and during hospitalization AFB: acid-fast bacilli, BAL: bronchoalveolar lavage, BUN: blood urea nitrogen, CSF: cerebrospinal fluid, HbA1c: hemoglobin A1c, IgG: immunoglobulin G, IgM: immunoglobulin M, PCR: polymerase chain reaction, WBC: white blood cell

Laboratory test	Value	Reference range
WBC count	18.5 × 10⁹/L	4.0-11.0 × 10⁹/L
Sodium	124 mmol/L	136-145 mmol/L
Chloride	92 mmol/L	98-107 mmol/L
BUN	21 mg/dL	7-20 mg/dL
Creatinine	1.1 mg/dL	0.6-1.3 mg/dL
Glucose	233 mg/dL	70-99 mg/dL
HbA1c	>14%	<5.7%
Osmolality, calculated	259 mOsm/kg	275-295 mOsm/kg
Lactate	1.1 mmol/L	0.5-2.0 mmol/L
*Coccidioides* IgM	Positive	Negative
*Coccidioides* IgG	Positive	Negative
BAL PCR for *Coccidioides*	Positive	Negative
AFB stain	Negative	Negative
CSF *Coccidioides* serology	Negative	Negative

Initial chest imaging demonstrated a right middle lobe opacity and numerous bilateral pulmonary nodules. CT revealed a 7.3 × 5.4 cm wedge-shaped necrotic mass in the right middle lobe with at least 15 bilateral pulmonary nodules, initially raising substantial concern for primary or metastatic malignancy (Figure 1). The wedge-shaped morphology also raised consideration of pulmonary infarction or septic embolic disease. Other diagnostic considerations included tuberculosis, endemic fungal infection, and other granulomatous processes.

**Figure 1:**
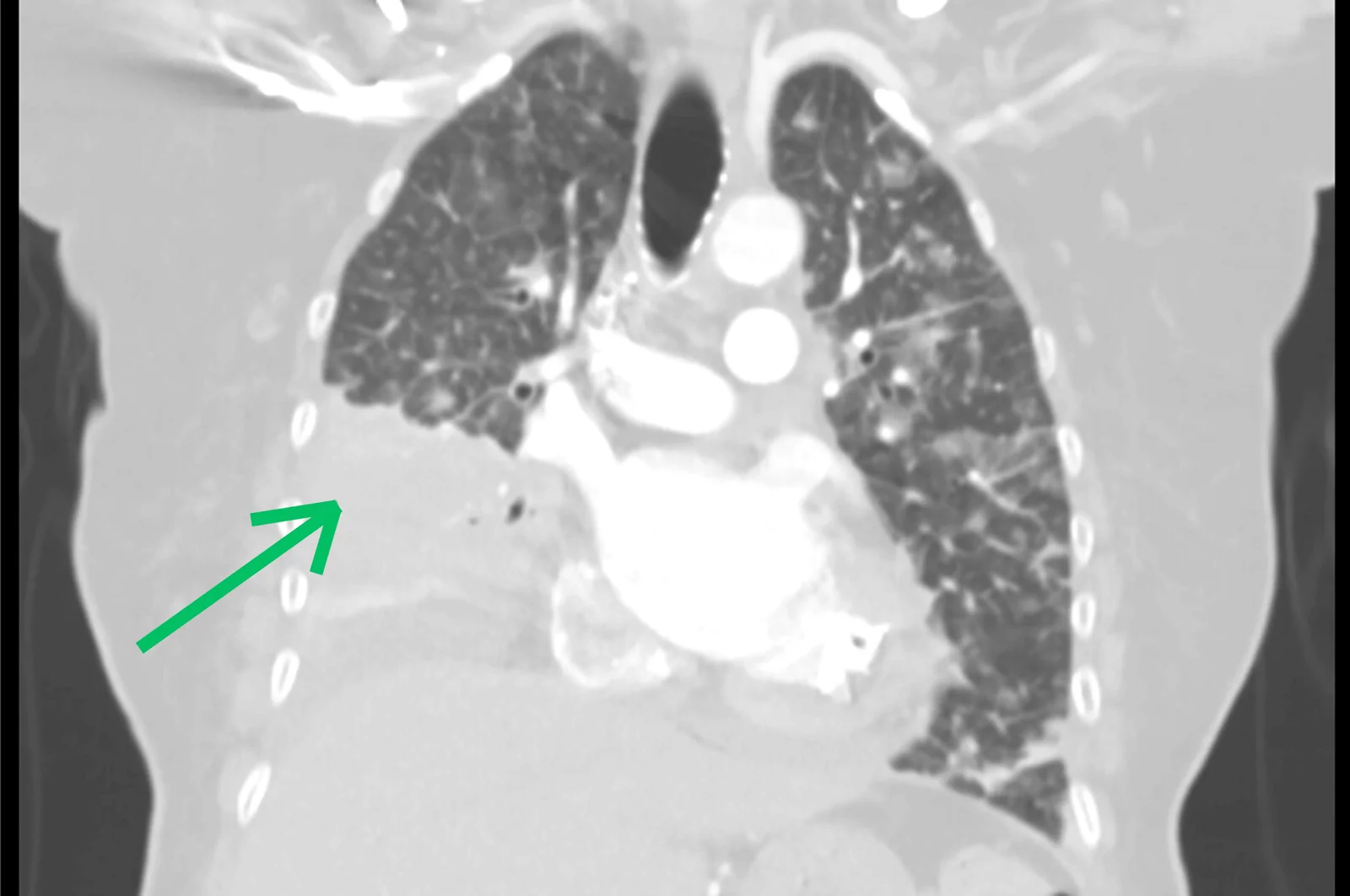
CT chest demonstrating wedge-shaped necrotic mass Contrast-enhanced CT scan of the chest showing a wedge-shaped necrotic mass in the right middle lobe (green arrow) with surrounding parenchymal opacities and diffuse bilateral pulmonary nodules, initially concerning for metastatic malignancy. CT: computed tomography

Intravenous piperacillin-tazobactam was initiated empirically on admission and discontinued on hospital day 5, following an infectious disease evaluation. Initial reference laboratory coccidioidal testing demonstrated an equivocal IgM result with negative complement fixation. Fluconazole 400 mg daily was initiated early in the hospitalization after coccidioidomycosis became a leading diagnostic consideration.

On hospital day 3, a CT-guided biopsy of the 7.3 × 5.4 cm right middle lobe mass demonstrated granulomatous inflammation with fungal elements on Grocott-Gomori methenamine silver staining, with no evidence of malignancy; acid-fast bacilli staining was negative. Subsequent *Coccidioides* immunodiffusion demonstrated IgG positivity, while complement fixation remained negative. A quantitative IgM titer was not reported. The evolution from an initially equivocal IgM result to IgG immunodiffusion positivity provided additional serologic support for infection, although complement fixation did not become positive.

Tuberculosis evaluation included QuantiFERON-TB Gold testing, which demonstrated a low mitogen response, as well as mycobacterial testing of respiratory specimens. *Mycobacterium tuberculosis* nucleic acid amplification testing from bronchial washings was negative. Serum galactomannan and β-D-glucan were not obtained. The pleural effusion was subsequently sampled by thoracentesis; cytology showed lymphocytes and macrophages without malignant cells.

Clinical course

By approximately hospital day 7, the patient developed progressive tachypnea, increasing work of breathing, worsening bilateral pulmonary infiltrates, and escalating oxygen requirements, requiring a high-flow nasal cannula. Her respiratory decline therefore began well before bronchoscopy, which was performed later after intubation.

Given rapidly progressive pulmonary disease, antifungal therapy was escalated from fluconazole to non-liposomal amphotericin B on hospital day 12. Despite maximal noninvasive support, the patient developed refractory hypoxemic respiratory failure and required endotracheal intubation and transfer to the intensive care unit on hospital day 15. At that time, she met Berlin criteria for severe ARDS, characterized by diffuse bilateral infiltrates, absence of cardiogenic pulmonary edema supported by echocardiography demonstrating preserved biventricular function, and a PaO₂/FiO₂ ratio <100.

Echocardiography demonstrated a left ventricular ejection fraction of 60-65%, normal right ventricular size and systolic function, mild mitral and tricuspid regurgitation, and no evidence of tamponade. B-type natriuretic peptide was 92 pg/mL, making a dominant cardiogenic mechanism for the bilateral pulmonary edema less likely.

She was managed with lung-protective mechanical ventilation using a tidal volume of approximately 230 mL, a positive end-expiratory pressure of 8 cm H₂O, and a documented plateau pressure of 23 cm H₂O. Because of persistent hypoxemia, early neuromuscular blockade was used from hospital days 16-18, and prone positioning was continued for approximately 48 hours.

Her renal function deteriorated rapidly after the initiation of amphotericin B. The acute kidney injury was clinically attributed primarily to amphotericin-associated acute tubular necrosis in the setting of concurrent critical illness, and hemodialysis was initiated on hospital day 16. Because of dialysis-requiring renal toxicity, non-liposomal amphotericin B was discontinued. Fluconazole was subsequently resumed at 400 mg twice daily on hospital day 26 because of suspected CNS infection.

Bronchoscopy with bronchoalveolar lavage (BAL) was performed on hospital day 17 after ARDS and mechanical ventilation were already established. Purulent secretions were noted in the right middle lobe. BAL testing subsequently returned positive for *Coccidioides* PCR, providing additional microbiologic confirmation of pulmonary infection.

After sedation had been weaned, the patient remained encephalopathic with limited responsiveness. Electroencephalography obtained off continuous sedation demonstrated diffuse cerebral slowing without evidence of an epileptiform process, consistent with a nonspecific global encephalopathy. Diffusion-weighted MRI revealed multiple foci of restricted diffusion involving gray-white matter junction regions, raising strong concern for an embolic or hematogenous process and, in the setting of proven pulmonary coccidioidomycosis, strongly suspected CNS dissemination (Figure 2).

**Figure 2:**
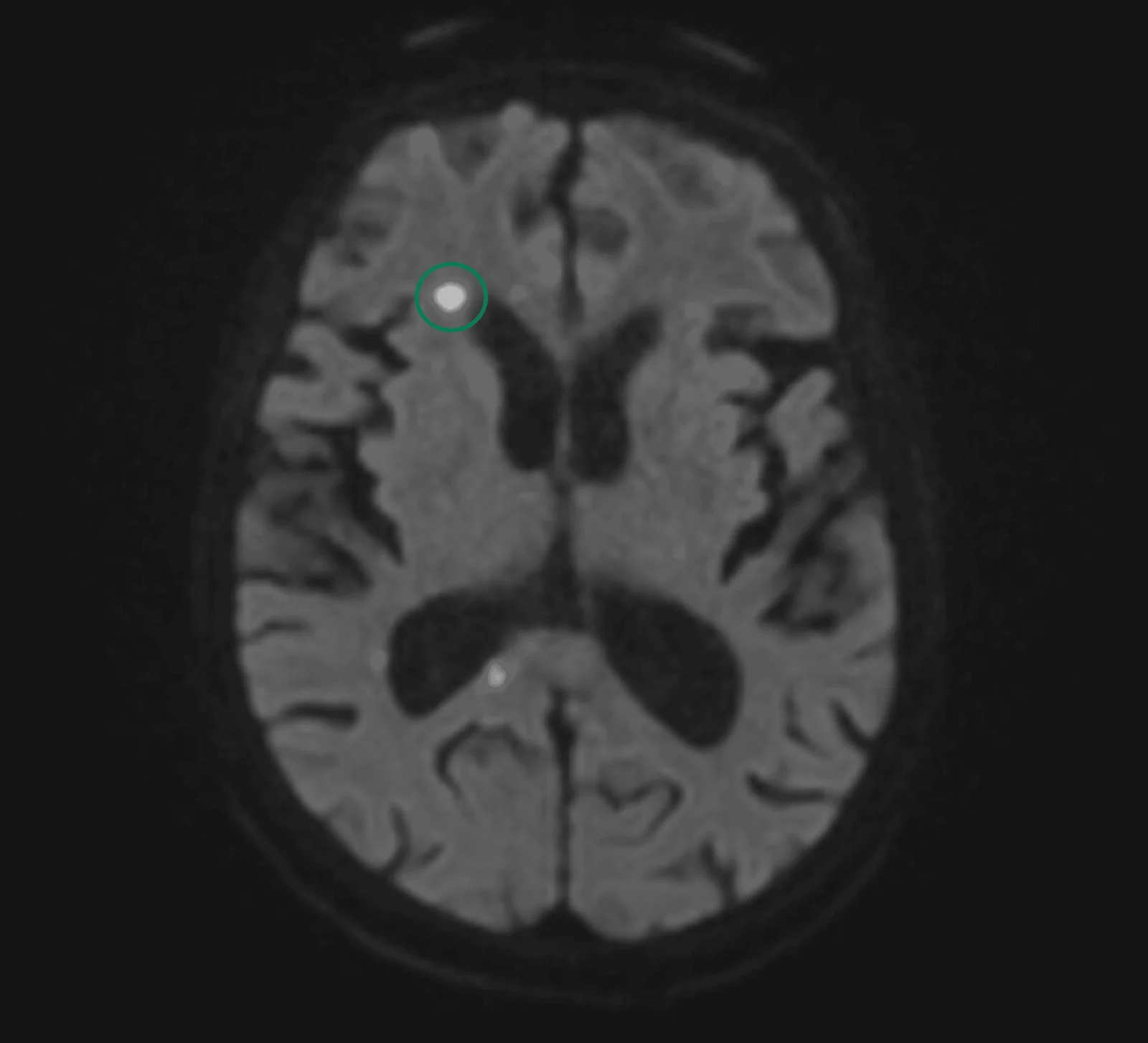
Diffusion-weighted MRI demonstrating focal restricted diffusion at the gray-white matter junction Axial diffusion-weighted MRI of the brain demonstrates a focal area of restricted diffusion (green circle) at the gray-white matter junction, concerning for embolic or hematogenous dissemination in the appropriate clinical context. MRI: magnetic resonance imaging

Lumbar puncture on hospital day 22 demonstrated normal opening pressure, a cerebrospinal fluid (CSF) white blood cell count of 2.75 cells/µL, protein 44 mg/dL, glucose 119 mg/dL, and negative *Coccidioides* IgM and IgG serology. Thus, the CSF profile did not establish coccidioidal meningitis. Nevertheless, because of the multifocal MRI abnormalities, persistent encephalopathy after sedation was discontinued, and severe pulmonary infection, neurology and the treating team maintained a high clinical suspicion for CNS dissemination. High-dose fluconazole at 400 mg twice daily was continued. Transesophageal echocardiography did not demonstrate intracardiac thrombus, septal defect, or endocarditis to provide an alternative cardiac source for the multifocal cerebral lesions.

The ICU course was further complicated by ventilator-associated *Klebsiella pneumoniae* pneumonia, treated with ampicillin-sulbactam from approximately hospital days 29-36. The patient also developed a right internal jugular vein thrombosis, considered catheter-associated, despite receiving subcutaneous heparin prophylaxis twice daily. Therapeutic anticoagulation with a heparin infusion was initiated but later limited by gastrointestinal bleeding in the setting of gastritis.

Because of prolonged ventilator dependence and inability to liberate from invasive respiratory support, tracheostomy was performed on hospital day 32, followed by percutaneous endoscopic gastrostomy placement on hospital day 33. The team held repeated goals-of-care discussions with the family throughout the prolonged hospitalization. Given the severity of illness and guarded prognosis, treatment options were reviewed repeatedly; the family elected to continue full disease-directed and life-prolonging treatment, and this preference was respected.

The patient later required ICU readmission around hospital day 45 for recurrent hypotension and developed new atrial flutter. No single definitive mechanism for the hemodynamic instability was established, and it was considered multifactorial in the setting of prolonged critical illness and infection. Despite ongoing supportive care, renal recovery was insufficient to discontinue renal replacement therapy.

By hospital day 54, she was hemodynamically stable enough for transfer to a long-term acute care facility. At discharge, she remained tracheostomy-dependent and dialysis-dependent, receiving hemodialysis three times weekly, and required ongoing respiratory care, enteral nutritional support, and rehabilitation.

## Discussion

This case illustrates an uncommon, severe manifestation of coccidioidomycosis, highlighting the intersection of infectious disease, pulmonary pathology, and critical care medicine.

Radiologic mimicry and the differential diagnosis of a wedge-shaped necrotic mass

Pulmonary coccidioidomycosis most often produces nodules, consolidation, adenopathy, or cavities, whereas a dominant mass-like lesion can closely mimic malignancy [5]. The wedge-shaped morphology in this case adds an important layer to the differential because peripheral wedge-shaped pulmonary abnormalities may also suggest infarction or septic embolic disease. The combination of diffuse bilateral nodularity, direct demonstration of fungal elements within the biopsied mass, subsequent *Coccidioides* serologic evidence, and positive BAL PCR ultimately favored coccidioidomycosis.

This diagnostic pathway illustrates a broader teaching point: in endemic regions, an atypical pulmonary mass should not automatically trigger a malignancy-only evaluation. Infectious and vascular diagnoses should be considered concurrently, and early tissue sampling may prevent prolonged diagnostic delay.

Epidemiology, host susceptibility, and multifactorial deterioration

The increasing reported burden of coccidioidomycosis in the United States likely reflects a combination of environmental conditions, population growth and migration into endemic regions, occupational and recreational soil exposure, and greater diagnostic awareness and surveillance [2,3]. Therefore, rising reported incidence should not be interpreted exclusively as a biological increase in infection.

Poorly controlled diabetes mellitus represented an important host susceptibility factor in this patient. Severe hyperglycemia can impair neutrophil chemotaxis and phagocytic function and compromise cell-mediated immune responses, potentially diminishing fungal clearance [6]. Infectious Diseases Society of America (IDSA) guidance also identifies extensive pulmonary involvement, diabetes mellitus, and significant comorbidity or frailty as factors that favor antifungal treatment [1].

At the same time, the severity of this hospitalization should not be attributed to coccidioidomycosis alone. Her deterioration was likely multifactorial, reflecting extensive pulmonary fungal disease superimposed on marked metabolic dysregulation, followed by ARDS, dialysis-requiring renal failure, ventilator-associated bacterial pneumonia, thrombosis, hemodynamic instability, and prolonged critical illness. This distinction matters in a single-case report, where associations can be described but direct causality cannot always be established.

ARDS in severe pulmonary coccidioidomycosis

Progression to ARDS represents a rare but catastrophic complication described in severe coccidioidomycosis, with published case series and observational data suggesting substantial morbidity and mortality [7]. In this patient, the temporal sequence is important. CT-guided biopsy of the right middle lobe mass was performed on hospital day 3, and progressive hypoxemia requiring high-flow nasal cannula developed approximately four days later. Her respiratory status continued to worsen, ultimately requiring intubation on hospital day 15. Bronchoscopy with BAL was performed on hospital day 17, after ARDS and invasive mechanical ventilation were already established, making a post-bronchoscopy inflammatory lung injury less likely as the primary explanation for her deterioration.

The pathophysiology of ARDS in severe fungal pneumonia likely reflects diffuse alveolar injury associated with a high pulmonary fungal burden and a dysregulated host inflammatory response, resulting in increased alveolar-capillary permeability and refractory hypoxemia. In this case, however, ARDS should be viewed as occurring in association with severe pulmonary coccidioidomycosis rather than as a definitively proven direct consequence, particularly given the contribution of critical illness and later nosocomial infection. Management followed established ARDS principles, including lung-protective ventilation with low tidal volumes, early neuromuscular blockade, and prone positioning to improve oxygenation and minimize ventilator-induced lung injury. Fungal-associated ARDS remains particularly challenging because pulmonary recovery may be delayed by persistent infectious burden and prolonged inflammatory injury.

CNS dissemination and diagnostic limitations

Neurologic involvement was strongly suspected but not microbiologically confirmed. Persistent encephalopathy after sedative medications were discontinued, along with multiple diffusion-restricting lesions at the gray-white matter junction, raised concern for hematogenous CNS dissemination.

Conversely, the lumbar puncture did not establish coccidioidal meningitis. Opening pressure was normal, CSF contained only 2.75 white blood cells/µL, protein was 44 mg/dL, and *Coccidioides* IgM and IgG were negative. Accordingly, the most accurate characterization is strongly suspected CNS dissemination rather than confirmed coccidioidal meningitis. CNS involvement remains the most severe form of dissemination. Negative CSF studies do not exclude early disease, and diagnosis often relies on clinical suspicion and imaging findings [4]. Encephalopathy in this setting was also plausibly multifactorial because renal dysfunction, prolonged critical illness, systemic infection, and prior sedative exposure may all contribute.

IDSA recommends CSF evaluation in patients with coccidioidomycosis who develop altered mental status or other concerning neurologic symptoms, and fluconazole 400-1200 mg daily is recommended as initial therapy for confirmed coccidioidal meningitis [1]. The patient's fluconazole dose of 400 mg twice daily therefore provided a CNS-penetrant azole regimen while neurologic involvement remained a major clinical concern.

Antifungal therapy and guideline-based management

The antifungal course reflected the changing severity of illness. Fluconazole was initiated early after coccidioidomycosis became the leading diagnosis, but with rapidly progressive pulmonary involvement and worsening respiratory failure, therapy was escalated to non-liposomal amphotericin B on hospital day 12. IDSA guidance supports amphotericin B for very severe or rapidly progressive pulmonary or disseminated coccidioidomycosis, followed by transition to an azole once clinically stable [1].

The course was complicated by acute kidney injury progressing to dialysis-requiring acute tubular necrosis, attributed primarily to amphotericin-associated nephrotoxicity, although concurrent critical illness may also have contributed. Amphotericin B was discontinued, and fluconazole was resumed at 400 mg twice daily. Lipid formulations of amphotericin B generally have a more favorable renal safety profile than conventional preparations, underscoring the need to balance efficacy with treatment-related toxicity.

Fluconazole remains the preferred initial azole for coccidioidal meningitis because of its favorable safety profile and CNS penetration. At the same time, itraconazole, voriconazole, and posaconazole may be considered in selected alternative or refractory cases [1]. In this patient, continued high-dose fluconazole provided systemic antifungal therapy and CNS penetration in the setting of strongly suspected neurologic dissemination.

Clinical learning points

Pulmonary coccidioidomycosis can present with atypical radiographic features, including necrotic lung masses that closely mimic malignancy, particularly in patients with underlying host susceptibility. Poorly controlled diabetes mellitus is a significant risk factor for dissemination and severe disease. In advanced cases, infection may progress to ARDS, requiring escalation to invasive mechanical ventilation and adjunctive strategies such as prone positioning and neuromuscular blockade. CNS involvement may occur despite negative CSF findings, necessitating a high index of clinical suspicion. Management requires careful balancing of antifungal efficacy and treatment-related toxicity, particularly in critically ill patients.

Limitations

This case is limited by the lack of definitive CSF confirmation of CNS involvement. However, clinical and radiographic findings strongly suggested CNS dissemination.

## Conclusions

Pulmonary coccidioidomycosis can present with atypical radiographic features, including necrotic lung masses that closely mimic malignancy. In endemic regions, unusual pulmonary lesions should prompt consideration of endemic fungal infection alongside malignancy and vascular processes, particularly in patients with impaired host defenses such as poorly controlled diabetes mellitus. Early recognition of the epidemiologic context and prompt tissue diagnosis are essential to avoid delays in appropriate therapy.

Severe coccidioidomycosis may reflect the convergence of host susceptibility, substantial fungal burden, and delayed recognition or treatment. In this patient, the disease course was complicated by ARDS, multisystem critical illness, and strongly suspected CNS dissemination despite negative CSF studies, underscoring the need for continued clinical vigilance when neurologic findings remain concerning.

Timely diagnosis and individualized antifungal therapy are critical, with careful attention to both treatment efficacy and toxicity. The central teaching point from this case is that atypical pulmonary imaging should always be interpreted in geographic and epidemiologic context, because failure to consider endemic fungal disease early may lead to diagnostic delay, progression of severe illness, and worse outcomes.
